# New York Letter

**Published:** 1895-07

**Authors:** J. L. Campbell

**Affiliations:** New York


### *CORRESPONDENCE.*

---

### NEW YORK LETTER.

NEW YORK, May 17, 1895.

*Editor Atlanta Medical and Surgical Journal:*

In writing a short letter to THE JOURNAL I will report a few cases I have seen in some of the hospitals here.

A man, aged about thirty years, had simple fracture of both bones of leg about juncture of middle and lower third. By this some of the deep veins were ruptured, which resulted in a vast extravasation of blood in the cellular tissues, extending up to the crest of the ilium, externally and posteriorly, to the perineum. The pressure was so great that gangrene was feared. Professor Dennis introduced a small trochar into the tissues and removed a small quantity of this blood, after which pulsations of the posterior and anterior tibial arteries were plainly felt, showing that they were not injured. The limb was again put up in the fracture-box and allowed to remain one week. When last seen the swelling was greatly reduced, pain had nearly ceased, and the pustular eruption, which was extensive a week ago, was nearly dried up. The limb was put up in plaster of Paris splint.

Professor Dennis says that he has treated several cases of this kind in like manner, and has invariably had good results. If you don't remove too much of the blood you will always be successful, for when you take some of the pressure off and give nature a chance she will do the work of absorbing the rest.

He also showed us a case of "black mole," or nevus, which he thinks ought to be always removed, especially those which have hairs growing in them, as they are likely to become malignant; and referred to a case he had only a few days ago, in which, on removing the mole, he found it had become malignant, although it showed no sign of cancer at the time.

A case of malignant tumor of the rectum removed at the polyclinic was very interesting, though I did not get the history. The tumor was situated about three inches from the anus and extended up nearly to the sigmoid flexure. The bowels were thoroughly moved and washed out, and, after the usual antiseptic preparations, an incision about four inches in length was made, beginning at the tip of the coccyx and extending up a little to the right of the median line. The coccyx and part of the sacrum were removed. The gut was surrounded and brought down into the wound. In doing this care was taken not to injure the vessels that supply the lower part of the rectum. It was about three or four inches in length and very thick. The operator then made an end to end anastomosis, holding the upper part of the rectum down by a silk ligature put in through the skin; this was to prevent too much tension on the part and prevent it pulling apart. The wound was dressed open and the patient put to bed. He did very nicely, and three weeks after the operation was in good condition, bowels moving regularly. The only trouble was an inability to pass urine, though he had the desire.

Another interesting feature of this case was the examination of the tumor. The pathologist examined it immediately and was able to tell the surgeon what it was before he got the patient dressed. And I think it a good idea for men to avail themselves of this plan when they are away from cities and are unable to have the more costly and troublesome apparatus. All you need is the double knife, or microtome, which can be carried in case and is no larger than a pair of scissors. It is two blades so arranged that they can be set so as to cut a section of any thickness you may want, and one very thin can be made when you have your blades sharp. Then you need a slide and cover-glass to remove the section which has been cut from the tumor, by pressing the microtome in the part from which you wish the section into a glass of water, and mount it. You can detect the cells very well, even in the unstained section, when the light is turned nearly off of the lens. This tumor proved to be an adenocarcinoma. Patient is able to walk around room, sit down, and is generally comfortable.

J. L. CAMPBELL, M.D.

